# Voltammetric Behaviour of Sulfamethoxazole on Electropolymerized-Molecularly Imprinted Overoxidized Polypyrrole

**DOI:** 10.3390/s8128463

**Published:** 2008-12-18

**Authors:** Sabriye Perçin Özkorucuklu, Yücel Şahin, Güleren Alsancak

**Affiliations:** 1 Süleyman Demirel University, Faculty of Arts and Sciences, Department of Chemistry, 32260 Isparta, Turkey; E-Mails: percin@fef.sdu.edu.tr; guleren@fef.sdu.edu.tr; 2 Anadolu University, Faculty of Science, Department of Chemistry 26470, Eskişehir, Turkey

**Keywords:** Molecularly imprinted polymer, overoxidized polypyrrole, pencil graphite electrode, electropolymerization, sulfamethoxazole, sulfonamides

## Abstract

In this work, preparation of a molecularly imprinted polymer (MIP) film and its recognition properties for sulfamethoxazole were investigated. The overoxidized polypyrrole (OPPy) film was prepared by the cyclic voltammetric deposition of pyrrole (Py) in the presence of supporting electrolyte (tetrabutylammonium perchlorate-TBAP) with and without a template molecule (sulfamethoxazole) on a pencil graphite electrode (PGE). The voltammetric behaviour of sulfamethoxazole on imprinted and non-imprinted (NIP) films was investigated by differential pulse voltammetry (DPV) in Britton-Robinson (BR) buffer solutions prepared in different ratio of acetonitrile-water binary mixture, between the pH 1.5 and 7.0. The effect of the acetonitrile-water ratio and pH, monomer and template concentrations, electropolymerization cycles on the performance of the MIP electrode was investigated and optimized. The MIP electrode exhibited the best reproducibility and highest sensitivity. The results showed that changing acetonitrile-water ratio and pH of BR buffer solution changes the oxidation peak current values. The highest anodic signal of sulfamethoxazole was obtained in BR buffer solution prepared in 50% (v/v) acetonitrile-water at pH 2.5. The calibration curve for sulfamethoxazole at MIP electrode has linear region for a concentration range of 25.10^-3^ to 0.75 mM (R^2^=0.9993). The detection limit of sulfamethoxazole was found as 3.59.10^-4^ mM (S/N=3). The same method was also applied to determination of sulfamethoxazole in commercial pharmaceutical samples. Method precision (RSD<1%) and recoveries (>87%) were satisfactory. The proposed method is simple and quick. The polypyrrole (PPy) electrodes have low response time, good mechanical stability and are disposable simple to construct.

## 1. Introduction

Sulfonamides have been used as antibacterial agents for last 60 years. A number of them have found widespread use in animal husbandry and, to a lesser extent, in the treatment of human infections such as bronchitis and urinary tract infections. The use of sulfonamide drugs in veterinary care without a proper withdrawal period will cause accumulation of sulfonamides in meat, eggs and milk as well as in fish. Because of the possible risk of resistance development in humans, the European Union (EU) has set a maximum residue limit (MRL, 0.1 μg/g ) for sulfonamides of animal originated products [1-2].

These low concentration limits led to development of fast and sensitive method to screen foodstuffs for sulfonamide based drugs. Several techniques have been reported in the literature for the determination of sulfonamides, i.e. gas chromatography (GC) and gas chromatography-mass spectrometry (GC-MS) [3], high performance liquid chromatography [4-7] and capillary electrophoresis [8-9] using various detectors. However there are only a few reports available on the electrochemical properties of sulfonamides [10-12].

Over the past two decades, molecular imprinted polymers (MIPs) have attracted a broad interest from scientists engaged in sensor development. This can be explained by the serious potential advantages of using MIPs in place of natural receptors and enzymes for their superior stability, low cost and easy preparation. The general principal of molecular imprinting is based on a process where functional and cross-linking monomers are copolymerization in presence of a target analyte (the imprint molecule) which acts as a molecular template. This procedure can be accomplished via either reversible covalent bonding or non-covalent interactions between monomers and imprint molecules. Other preparation methods of molecular imprinting polymers are chemical grafting, soft lithography technique [13], molecular self-assembled approach [14] and electropolymerization [13]. These films can also be synthesized *in situ* at an electrode surface by electropolymerization technique. This technique has some attractive features including the easy adherence of the polymeric films to the surface of conducting electrodes of any shape and size and the ability to control thickness of the films under different depositions conditions [15]. Various types of electrosynthesized polymers based on molecular imprinting have been reported in the literature including poly(*o*-phenylenediamine) [15], poly(2-mercaptobenzimidazole) [16], polypyrrole [17], polyphenol [18] and copolymer of aniline with *o*-phenylendiamine [19].

**Scheme 1.**
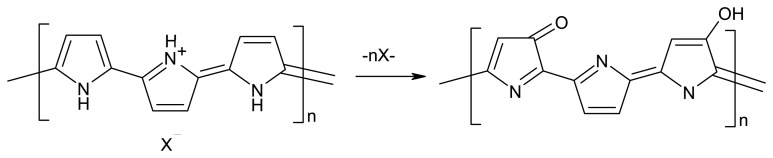
The overoxidation reaction of polypyrrole

Among various types of conducting polymers, polypyrrole has many attractive features as a molecular recognition system, since it can be used in a neutral pH region, and its stable films can conveniently be polymerized on various substrate materials. Sensors based on molecularly imprinted polypyrrole for amino acids [20], caffeine [21-23], paracetamol [24], ascorbic acid [25], isoniazid [26], glycoproteins [27] were reported. Our group prepared a molecularly imprinted polypyrrole-based films for the determination of paracetamol and ascorbic acid. The performance of the imprinted films was evaluated by differential pulse voltammetry. This film exhibited a high selectivity and sensitivity toward paracetamol and ascorbic acid [24-25]. PPy undergoes overoxidation at positive potentials, and this process has often been regarded as an undesirable degradation process, which leads to the loss of conductivity and dedoping [28]. Despite these disadvantages, OPPy has been used in some electroanalytical applications; the overoxidized film works as a porous electrode coating, which has cation-exchange and molecular sieve properties [20, 26, 29-33]. It has been reported that, during oxidation polypyrrole loses its electroactivity due to ejection of dopant, and oxygen-containing groups such as carbonyl and carboxyl are introduced to the pyrrole unit [34]. The accumulative properties of the film for cationic species might be attributed to the introduction of carboxylate. The best results are achieved if during electrochemical deposition OPPy is imprinted by small molecular weight molecules [21-22]. Moreover, attempts to imprint PPy by large molecular weight rigid structure possessing proteins were reported as well as, in this case viral envelope proteins possessing rigid structure were imprinted within OPPy [27]. PPy is overoxidized chemically or electrochemically to cause dedoping as shown in Scheme 1.

**Figure 1.**
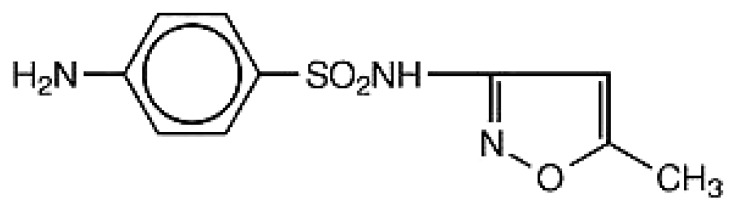
The structure of sulfamethoxazole

The use of a differential pulse voltammetry to determine the sulfamethoxazole using pencil graphite electrode coated with overoxidized polypyrrole films by imprinting electropolymerization was reported for the first time in this work. The structure of sulfamethoxazole is shown in Figure 1. Its successful application for determination of this drug in pharmaceutical sample has been demonstrated.

## 2. Results and Discussion

### 2.1. Electropolymerization of molecularly imprinted overoxidized polypyrrole

**Figure 2.**
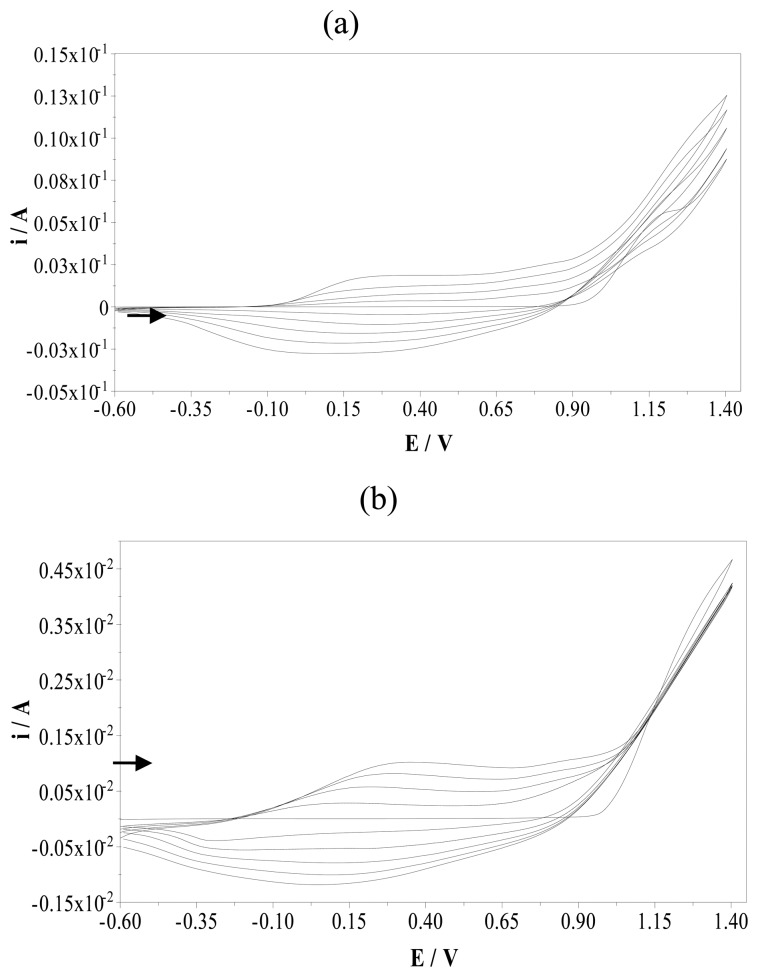
Cyclic voltammograms taken during the electropolymerization of pyrrole (0.1 M). (a) Multisweep cyclic voltammograms without and (b) with sulfamethoxazole (5 mM) onto a pencil graphite electrode (scan rate: 100 mVs^-1^; supporting electrolyte: 0.1 M TBAP; number of scans: 5)

The electrochemical behaviour of pyrrole was investigated in acetonitrile solution of 0.1 M TBAP using potential cycling between -0.6 and +1.40 V (versus Ag/AgCl) with PGE. Electrooxidation of pyrrole monomer occurs at the anode and the resulting polymer deposits onto the surface of PGE. An anodic peak of pyrrole was observed at a peak potential of 1.20 V. The corresponding reduction process was not observed on the cyclic voltammogram. The oxidation peak corresponds to the formation of pyrrole radical cations. Figure 2a demonstrates five cycles obtained in the same solution. The formation and growth of the polymer film can be easily seen in this figure. The peaks due to the oxidation and reduction of the film increase intensity as the film grows. A broad oxidation peak was observed at the peak potential of +0.15 V and reverse cathodic peak was seen at a peak potential of -0.10 V.

For imprinted electropolymerizations, 5 mM sulfamethoxazole as a template was added to the electrochemical cell. Figure 2b demonstrates the cyclic scans of electropolymerization of pyrrole in the presence of sulfamethoxazole. The effect of sulfamethoxazole on the electropolymerization of pyrrole can be seen easily in this figure. Even though sulfamethoxazole is an electro-inactive template, the oxidation peak potential of polypyrrole shifted to more anodic potentials, from 0.15 to 0.35 V, and approximately ten times smaller oxidation peak current value was observed. This oxidation peak indicates that the template is becoming part of the polymeric chain [17]. Sulfamethoxazole diffuses towards the surface of PGE entrapped in the polymer matrix during polymerization process of pyrrole. This is the key process towards achieving a voltammetric sensor based on molecular imprinting polymers. The creation of the molecular imprint is favored by the diffusion of the electroactive template, generating a far higher number of recognition sites than those previously obtained with non-electroactive template. If the template is non-electroactive, molecular diffusion towards the electrode is interrupted after the first scans by the formation of the non-conductive polymeric layer, which prevents the template forming part of this layer and thereby decreasing the peak current intensity.

**Figure 3.**
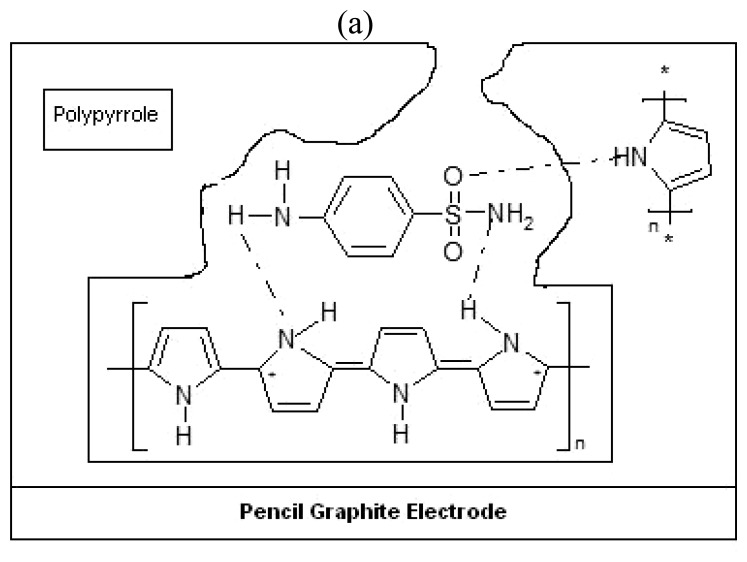

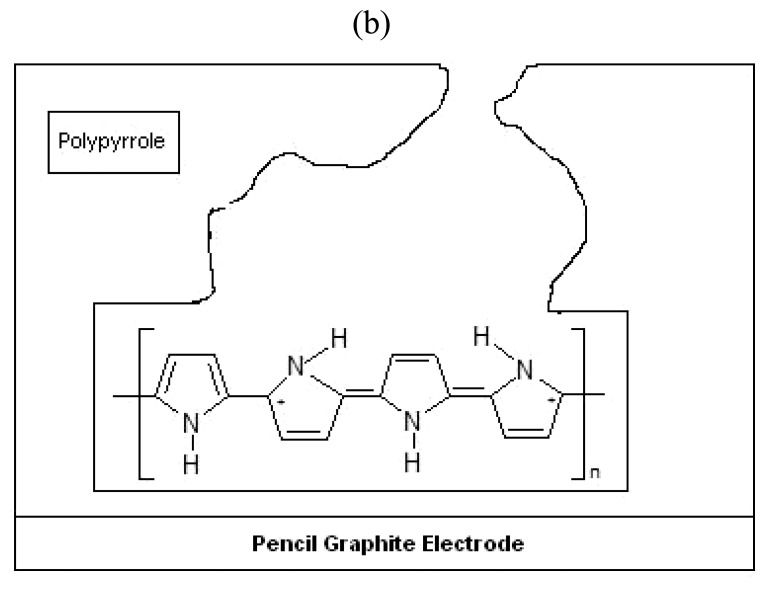
Schematic representation of **(a)** imprinting and **(b)** removal of sulfamethoxazole from sulfamethoxazole imprinted polypyrrole modified pencil graphite electrode.

During the electrodeposition of pyrrole, sulfamethoxazole template molecules are trapped in to the polymer matrix as a result of the ability of these molecules to interact with the pyrrole units. Figure 3. shows a schematic representation of imprinting and removal of sulfamethoxazole from sulfamethoxazole imprinted polypyrrole modified pencil graphite electrode. The oxygen atom in the S=O group of the sulfamethoxazole molecule forms a hydrogen bond with the hydrogen atom in the N-H group of the pyrrole units (Figure 3). Hydrogen bonding could occur between the hydrogen in the amino group of sulfamethoxazole structure and the nitrogen atom of the pyrrole N-H groups. Chain branching and cross linking in polypyrrole generate a three-dimensional matrix with niches containing the template sulfamethoxazole. This imprinting process creates a microenvironment for the recognition of sulfamethoxazole molecule based on shape selection and positioning of the functional groups.

In order to remove the entrapped template, various strategies are possible: microwave assisted extraction, supercritical fluid desorption, oxidation-reduction of template in polymer, the use of solvent that strongly interacts with polymer causing the swelling of the coating necessary for the template release or overoxidation. To remove the template inside the polypyrrole, overoxidation process was used in our work. The polymer was overoxidized by scanning the potential in a range of +0.80 to +1.20 V during twenty cycles (scan rate: 50 mV/s) in 0.1 M NaOH solution.

### 2.2. Effect of the acetonitrile-water ratio and pH

**Figure 4.**
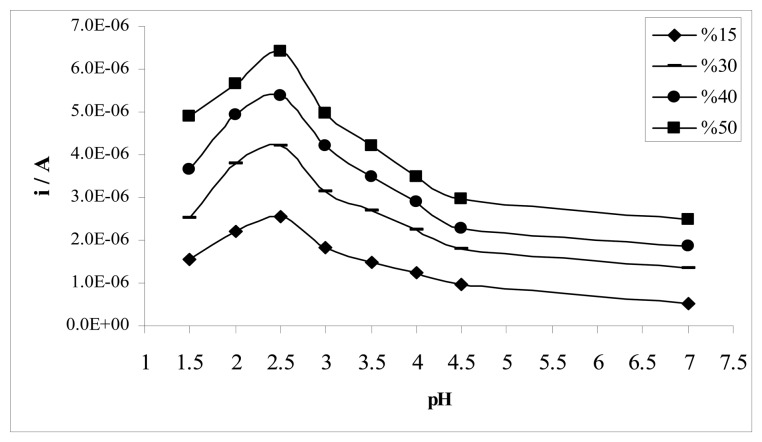
Effects of acetonitrile-water ratio and pH on the MIP modified PGE response for 0.10 mM sulfamethoxazole in Britton-Robinson buffer solution.

The mechanism for electrochemical oxidation of sulfamethoxazole depends on the acetonitrile-water ratio and pH of the solution. The effects of acetonitrile-water ratio and pH of the solution on the MIP electrode is illustrated in Figure 4 by DPV. The highest peak current intensity was observed in %50 (v/v) acetonitrile-water Britton-Robinson buffer at pH 2.5, giving an oxidation peak at 1.15 V.

### 2.3. Electrochemical behaviour of sulfamethoxazole

**Figure 5.**
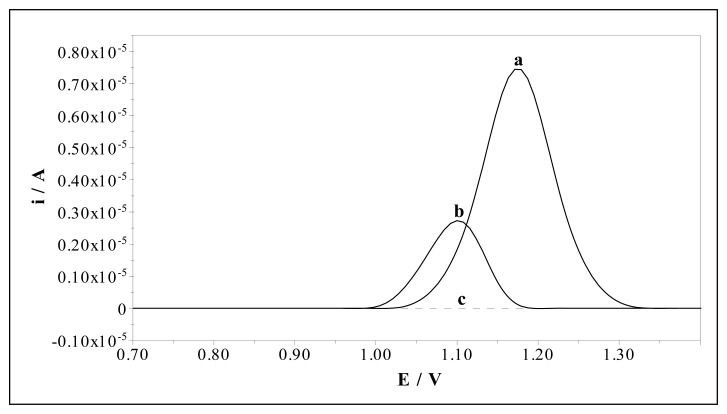
Differential pulse voltammograms for (a) 0.10 mM sulfamethoxazole at MIP electrode (b) 0.10 mM sulfamethoxazole at NIP electrode and (c) blank solution at MIP electrode in 50% (v/v) acetonitrile-water Britton-Robinson buffer at pH 2.5.

The electrochemical behaviour of sulfamethoxazole (0.10 mM) was investigated by MIP and NIP electrodes in 50% (v/v) acetonitrile-water BR buffer at pH 2.5 by DPV. The catalytic effect of the MIP electrode is demonstrated in Figure 5. Sulfamethoxazole gives an oxidation peak response at about 1.08 V and 1.15 V (versus Ag/AgCl) at the NIP and MIP electrodes, respectively. The enhanced peak current response and a shift in the oxidation potential of sulfamethoxazole by about 70 mV are a clear evidence of the catalytic effect of the MIP electrode towards the oxidation of sulfamethoxazole.

**Figure 6.**
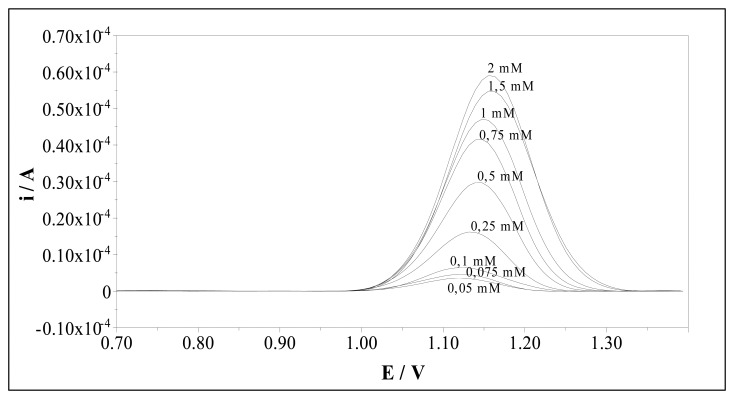
Differential pulse voltammograms of varying concentration at the MIP electrode at pH 2.5.

**Figure 7.**
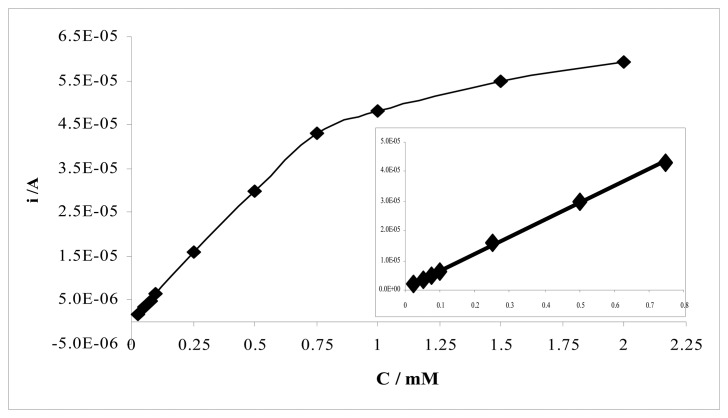
The calibration curves of sulfamethoxazole between peak current and concentration of sulfamethoxazole at MIP electrode.

The voltammetric determination of sulfamethoxazole using MIP and NIP electrodes results in well-defined concentration dependences. Figure 6 displays differential pulse voltammograms for solutions containing increasing quantities of sulfamethoxazole at the MIP electrode. The calibration curve of sulfamethoxazole at MIP electrode at pH 2.5 was given in Figure 7. Two linear regions were observed between peak current and concentration of sulfamethoxazole for MIP electrode. The first region demonstrates linearity over a concentration range of 25.10^-3^ mM to 0.75 mM with a correlation coefficient of 0.9993. The slope of the second linear region in the concentration range of 0.75-2.0 mM is smaller than the first region's slope. The correlation coefficient of this region was 0.992. Table 1 represents calibration characteristics and related parameters for sulfamethoxazole using MIP electrode. The limit of detections (LOD) and limit of quantification (LOQ) of sulfamethoxazole with MIP electrode were calculated according to the 3*s*/*m* and 10*s*/*m* criterious, respectively. The results are also shown in Table 1.

### 2.4. Effect of the monomer concentration

**Figure 8.**
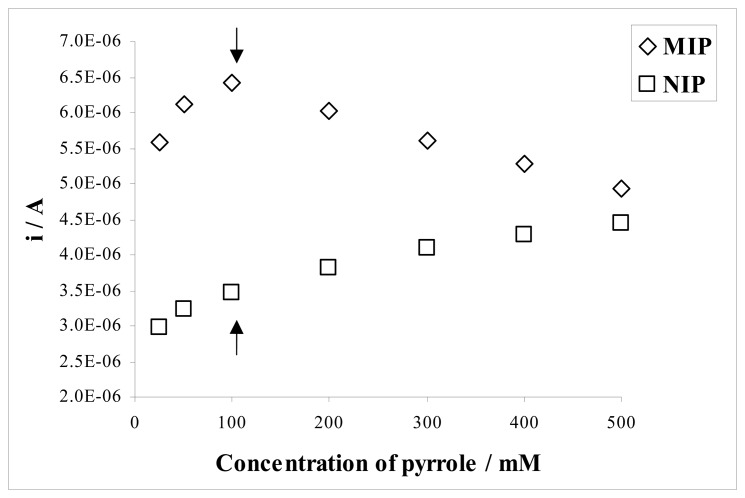
Effect of the monomer concentration on (Δ) MIP and (□) NIP electrodes for 0.10 mM sulfamethoxazole in 50% (v/v; acetonitrile-water Britton-Robinson buffer at pH 2.5.

The monomer concentration during polymerization also determined the analytical behaviour of the sensor. To determine the effect of monomer concentration on the response of both MIP and NIP to sulfamethoxazole, the films were grown in solutions of constant concentration of sulfamethoxazole and varying pyrrole concentrations in the range of 25-500 mM by cycling potential between -0.60 V and +1.40 V. Figure 8 shows the variation of the monomer concentration as a function of the current values for sulfamethoxazole. The monomer concentration should be proportional to the thickness of the deposit and amount of imprinted molecule (template) in the polymer matrix. The response of the MIP electrode to sulfamethoxazole was found to increase with increasing pyrrole concentration up to 100 mM. There was considerable decrease in the response of MIP electrode below and above this pyrrole concentration. The current difference between the NIP and NIP electrodes for sulfamethoxazole should be as high as possible. In addition, the signal of the NIP electrode should be a minimum. It can be concluded that the optimum monomer concentration under these conditions was about 100 mM as clearly seen in Figure 8.

### 2.5. Effect of the electropolymerization cycles

**Figure 9.**
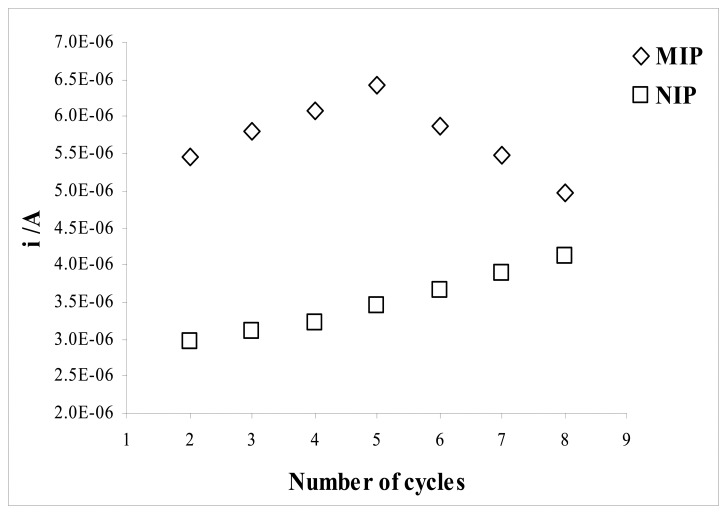
Effect of the number cycle to (Δ) MIP and (□) NIP electrodes for 0.10 mM sulfamethoxazole in 50% (v/v; acetonitrile-water Britton-Robinson buffer at pH 2.5.

The optimum number of CV cycles to use to form the sensing layer of the electrode was determined from a series of experiments in which electrodes were fabricated with different numbers of cycles (Figure 9).

The number of cycles applied to the cell during the electropolymerization was found to affect the sensitivity and linearity of the sensor. The MIP electrode produced at lower number of cycles exhibited favorable analytical performance. Higher cycles lead to more extensive electropolymerization, and therefore to the formation of thicker sensing film with less accessible imprinted sites. The response of the NIP electrode to sulfamethoxazole was found to increase with increasing the number of cycles. There was considerable decrease in the performance of the MIP electrode below and above five cycles. The highest current difference between the MIP and NIP electrodes for sulfamethoxazole was obtained by applying five cycles in the electropolymerization. Therefore the optimum polymerization cycles was found to be five.

### 2.6. Effect of the template concentration

**Figure 10.**
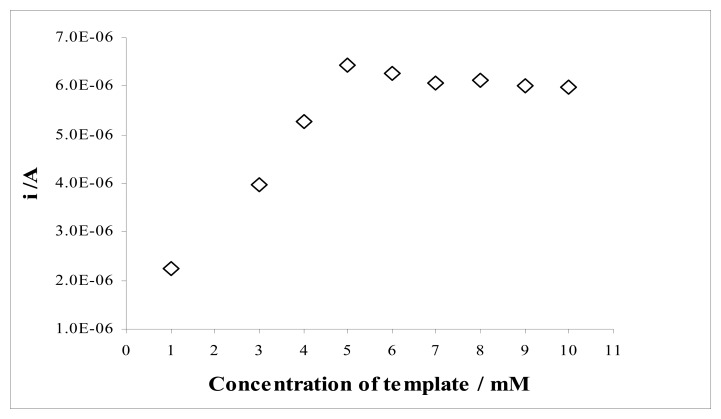
Effect of the template concentration to MIP response.

The template concentration at the moment of electropolymerization may also be significant factor in the response of the sensor. Figure 10 shows the effect of the template concentration during electrodeposition of the film in the range of 1.0-10 mM. The response of the MIP electrode to sulfamethoxazole increases with the increase of the template concentration between 1.0 and 5.0 mM. There was a small decrease in the response of MIP electrode above this sulfamethoxazole concentration (5.0 mM). Based on the results, the optimum template concentration was chosen as 5.0 mM.

### 2.7. Analysis of pharmaceutical samples

In order to demonstrate the practical usage of the biosensor, tablet and syrup having sulfamethoxazole was examined for estimation of sulfamethoxazole by MIP electrode. Solution obtained by dissolution of sulfamethoxazole tablet and syrup were subsequently diluted so that sulfamethoxazole concentration lies in the range of calibration plot. Differential pulse voltammograms were then recorded under exactly identical conditions that were employed while recording differential pulse voltammograms for plotting calibration plot. The amounts of sulfamethoxazole that reported and experimentally determined in tablet and syrup are listed in Table 2. It was found that sulfamethoxazole concentration determined using this method is in good agreement with the manufacturer's stated contents of sulfamethoxazole. To validate the voltammetric detection method, the pharmaceutical samples were also determined with a HPLC method at the optimal chromatographic conditions. Table 2 shows the obtained data from the HPLC and DPV with the MIP electrode. The all values in this table are mean of five replicate measurements. The results reveal that both methods had adequate precision and accuracy. Therefore they both can be applied to the determination of sulfamethoxazole in pharmaceutical formulations.

A Student *t*-test and *F*-test were carried out on the data to statistically examine the validity of the obtained results. At 95% confidence level, the calculated *t* and *F* values were less than of theoretical *t* and *F* values showing that there is no significative differences between the DPV and HPLC methods. The developed DPV method is simpler, faster and requires less expensive equipment than chromatographic methods.

### 2.8. Reproducibility of the MIP electrode

The reproducibility of the molecularly imprinted modified pencil graphite electrode was investigated for 0.10 mM sulfamethoxazole. The peak current response of sulfamethoxazole was determined with five electrodes which produced under the same conditions. The response peak intensity showed a relative standard deviation of 3.4% confirming that the results are reproducible.

## 3. Experimental Section

### 3.1. Chemicals and reagents

Sulfamethoxazole (>99.9%) was obtained from Sigma-Aldrich (Germany). Acetonitrile (>99.9%, Sigma-Aldrich, Germany), tetrabutylammonium perchlorate (99%, Fluka, Germany), sodium hydroxide (>98%, Merck, Germany), potassium hydrogen phthalate (>99.5%, Merck, Germany, dried at 110 oC before use), phosphoric acid (85%, Riedel-de Haen, Germany), acetic acid (Glacial, Riedel-de Haen, Germany), boric acid (>99.5%, Carlo Erba, Italy) are commercially available as analytical grade reagents. Pyrrole (98%, Aldrich, Germany) was distilled repeatedly under vacuum until a colorless liquid was obtained and kept under nitrogen in darkness at 4°C. Ultra-pure deionized water (Sartorius), with a resistivity of 18.2 MΩ cm, was used for all experiments. Trimoks Fort tablet (Atabay) and Bactrim syrup (Roche) were purchased from a local pharmacy. Freshly prepared solution of sulfamethoxazole was prepared each day owing to its low stability.

### 3.2. Apparatus

All electrochemical studies were carried out with Autolab PGSTAT 100 Potentiostat/Galvanostat controlled by GPES 4.9 version software (Ecochemie, The Netherlands). The three-electrode system was used for all measurements; a pencil graphite electrode (PGE) as the working electrode, a Pt auxiliary electrode and an Ag/AgCl reference electrode. MA 235 model (Mettler Toledo, Switzerland) pH-Ion meter with InLab 416 Ag/AgCl glass electrode was used for pH measurements. Chromatographic measurements were carried out using a Agilent 1100 series HPLC (Germany) consisting of a gradient pump, a YMC Pack ODS-AM (5μm, 250 mm × 4,6 mm ID) end-capped column coupled with an UV-Vis detector and computer. The eluent was acetonitrile-water mixture (%50 (v/v)) in Britton-Robinson buffer (pH 2.5) at a flow rate of 1 mL·min^1^. All separation was carried out at 25°C. The detection was performed at 250 nm.

### 3.3. Preparation of solutions

The voltammetric behaviour of sulfamethoxazole was investigated at Britton-Robinson (BR) buffer solutions prepared in 15, 30, 40 and 50 % (v/v) acetonitrile-water binary mixture at pH value from 1.5 to 7.0. A stock BR buffer solution composed of mixture of boric, acetic and phosphoric acids (each 0.04 M) were prepared and its pH values were adjusted by the addition of 1.0 M NaOH. pH meter was calibrated in the acetonitrile-water solution with different hydro-organic mixtures potassium hydrogenphthalate buffers (0.05 mol·kg^-1^). A standard stock solution of sulfamethoxazole (10 mM) was obtained by dissolving 0.0253 g of sulfamethoxazole in a 10 mL volumetric flask for each acetonitrile-water mixture.

Two pharmaceutical tablets having sulfamethoxazole were weighed and powdered homogeneously in a mortar. A quantity of the powder, equivalent to one tablet, was dissolved in 50% acetonitrile-water mixture. The solution was sonicated for 10 min and filtered An aliquot of appropriate volume of stock solution was transferred into 250 mL volumetric flask and volume was completed with BR buffer (50% acetonitrile-water mixture, pH 2.5). The syrup was transferred to a 100 mL flask and volume was adjusted to the same pH value with buffer solutions. The samples were then spiked with appropriate amount of sulfamethoxazole for experiments.

### 3.4. Preparation of MIP and NIP electrodes

A Noki pencil model 2000 (Japan) was used as a holder for graphite leads (Tombo, HB, 0.5 mm diameter, Japan). The PGE was prepared by cutting the leads into 3 cm long sticks. Electrical contact with the lead was obtained by soldering a metal wire to the metallic part. PGEs were washed with acetonitrile to remove the impurity and dried at room temperature before use. Then, PGE was immersed the polymerization solution.

**Figure 11.**
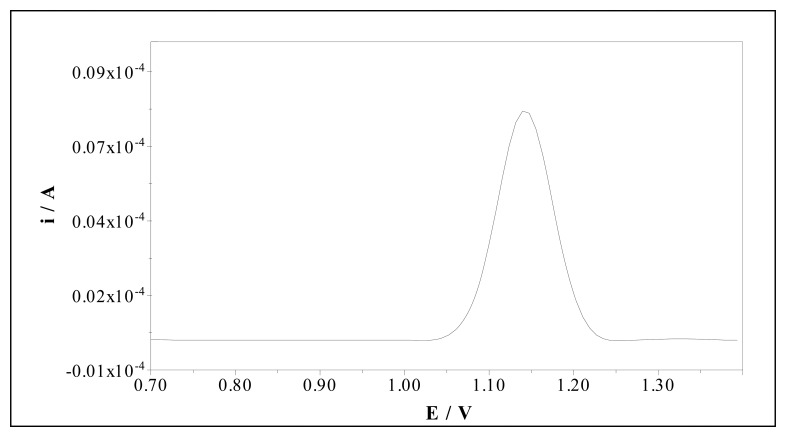
Differential pulse voltammogram of template by PGE in a desorption solution.

The MIP was obtained by electrodeposition on the surface of the PGE using cyclic voltammetry in the potential range between -0.60 and +1.40 V during five cycles (scan rate: 100 mV/s). The polymerization solution includes 0.1 M tetrabutylammonium perchlorate (TBAP), 0.1 M pyrrole and 0.005 M sulfamethoxazole in acetonitrile. After the electropolymerization process, the embedded sulfamethoxazole were then extracted to give a surface complimentary in shape and functionality to the original template sulfamethoxazole. The imprinted polymer was overoxidized in 0.1 M NaOH solution. The overoxidation was performed by scanning the potential repeatedly in a range of +0.80 to +1.20 V at a scan rate of 50 mV/s. We determined the sulfamethoxazole in this solution using DPV by uncovered PGE. Figure 11 shows that the template extracted from the polymer transferred in to this solution. A control electrode (non-imprinted overoxidized polypyrrole modified electrode, OPPY-NIP) was prepared in every case under the same experimental conditions but without adding the sulfamethoxazole, to check the reliability of the measurements.

### 3.5. Electroanalytical measurements

Voltammetric measurements were carried out in three-electrode cell, at Britton-Robinson (BR) buffer solutions prepared in 15, 30, 40 and 50% (v/v) acetonitrile-water binary mixture from pH 1.5 to 7.0. Before the measurements, electrolytic solutions were purged with nitrogen for 5 min. Current measurements were performed using differential pulse voltammetry in a potential range between 0.00 and 1.40 V at a scan rate of 15 mV s^-1^, modulation amplitude of 50 mV and step potential of 8 mV. All electroanalytical measurements were made at room temperature.

## 4. Conclusions

In this study, sulfamethoxazole imprinted electrodes formed by the cyclic voltammetric deposition of polypyrrole film on pencil graphite electrode in the presence of sulfamethoxazole have been fabricated. The molecularly imprinted electrode has been successfully applied as a sensor for fast and accurate determination of sulfamethoxazole in standards and pharmaceutical samples. The molecularly imprinted film exhibited a high sensitivity to sulfamethoxazole in 50% (v/v) acetonitrile-water mixture at pH 2.5. A linear relationship between sulfamethoxazole concentration and current response was obtained with excellent reproducibility of the current and a low detection limit of 3.59.10^-7^. The result of limit detection is lower than that reported in the literature using HPLC [6] and capillary electrophoresis [8] methods. Additionally, compared with results on application of molecularly imprinted polymer reported by other authors [16, 19, 21], the detection limit determined using MIP-based sensor indicate no significant difference.

The MIP based sensor was used for determination of sulfamethoxazole in commercial pharmaceutical samples. The recoveries in tablet and syrup were found as 96.0 and 87.4 % with the relative standard deviations of 0.90 and 0.85%, respectively. The sulfamethoxazole concentrations determined using this method is in good agreement with declared values. The proposed low cost chemical sensor could find application in the measurement of sulfamethoxazole level in clinical samples as well as in pharmaceutical industry.

## Figures and Tables

**Table 1.** Characteristics of sulfamethoxazole calibration plots.

	**Sulfamethoxazole**	
Linearity range (mM)	25.10^-3^- 0.75	0.75-2.0
Slope	5.75.10^-5^	1.28.10^-5^
Intercept	7.15.10^-7^	3.45.10^-5^
Correlation coefficient	0.9993	0.9920
RSD of slope	1.08	0.98
RSD of intercept	0.77	1.81
LOD	3.59.10^-4^ mM	
LOQ	1.20.10^-3^mM	

**Table 2.** Determination of sulfamethoxazole in pharmaceutical preparations using DPV at molecularly imprinted polypyrrole modified pencil graphite electrode and HPLC.

	DPV		HPLC	

	Trimoks Fort Tablet	Bactrim Syrup	Trimoks Fort Tablet	Bactrim Syrup
Reported Content	0.250^a^	50^b^	0.250^a^	50^b^
Detected Content	0.240^a^	43.7^b^	0.245^a^	47.4^b^
Recovery%	96.0	87.4	98.0	94.8
RSD%	0.90	0.85	1.08	0.76
Student t-test	0.103	0.379	0.420	0.160
F-test	1.500	1.063	*t*_theoretical_ : 2.31	*F*_theoretical_: 6.39

a g/tablet

b mg/100 mL

## References

[1] Zheng N., Li Y., Wen M. (2004). Sulfamethoxazole-imprinted polymer for selective determination of sulfamethoxazole in tablets. J. Chromatogr. A.

[2] Commission of the European Community (1991). The rules governing medical products in the European Community VI..

[3] Reeves V.B. (1999). Confirmation of multiple sulfonamide residues in bovine milk by gas-positive chemical ionization mass spectrometry. J. Chromatogr. B.

[4] Klimes J., Mokry M. (1997). High performance liquid chromatography analysis of selected sulfonamides in plasma. Pharmazie.

[5] Stoev G., Michailova A. (2000). Quantitative determination of sulfonamide residue in foods of animal origin by high-performance liquid chromatography with fluorescence. J. Chromatogr. A.

[6] Akay C., Özkan S.A. (2002). Simultaneous LC determination of trimethoprim and sulphamethoxazole in pharmaceutical formulations. J. Pharm. Biomed. Anal..

[7] Zayas-Blanco F., Garcia-Falcon M.S., Simal-Gandara J. (2004). Determination of sulfamethazine in milk by solid phase extraction and liquid chromatographic separation with ultraviolet detection. Food Control.

[8] Berzas Nevado J.J., Castaneda Penalvo G., Guzman Bernardo F.J. (2001). Determination of sulfametoxazole, sulfadiazine and associated compounds in pharmaceutical by capillary zone electrophoresis. J. Chromatogr. A.

[9] Fuh M.S., Chu S. (2003). Quantitative determination of sulfonamide in meat by solid-phase extraction and capillary electrophoresis. Anal. Chim. Acta.

[10] Rao T.N., Sarada B.V., Tryk D.A., Fujishima A. (2000). Electroanalytical study of sulfa drugs at diamond electrodes and their determination by HPLC with amperometric detection. J. Electroanal. Chem..

[11] Msagati T.A.M., Ngila J.C. (2002). Voltammetric detection of sulfonamides at a poly(3-methylthiophene) electrode. Talanta.

[12] Preechaworapun A., Chuanuwatanakul S., Einaga Y., Grudpan K., Motomizu S., Chailapakul O. (2005). Electroanalysis of sulfonamides by injection system/high-performance liquid chromatography coupled with amperometric detection using boron-doped diamond electrode. Talanta.

[13] Haupt K. (2001). Molecularly imprinted polymers in analytical chemistry. Analyst.

[14] Piletsky S.A., Piletskaya E.V., Sergeyeva T.A., Panasyuk T.L., El'skaya A.V. (1999). Molecularly imprinted self-assembled films with specificity to cholesterol. Sens. Actuat. B.

[15] Malitesta C., Losito I., Zambonin P.G. (1999). Molecularly imprinted electrosynthesized polymers: New materials for biomimetic sensors. Anal. Chem..

[16] Gong J., Gong Fu., Zeng G., Shen G., Yu R. (2003). A novel electrosynthesized polymer applied to molecular imprinting technology. Talanta.

[17] Ramanavicius A., Ramanaviciene A., Malinauskas A. (2006). Electrochemical sensors based on conducting polymer-polypyrrole. Electrochim. Acta.

[18] Panasyuk T.L., Mirsky V.M., Piletsky S.A., Wolfbeis O.S. (1999). Electropolymerized molecularly imprinted polymers as receptor layers in capacitive chemical sensors. Anal. Chem..

[19] Gomez-Caballero A., Goicolea M.A., Barrio R.J. (2005). Paracetamol voltammetric microsensors based on electrocopolymerized-molecularly imprinted film modified carbon fiber microelectrodes. Analyst.

[20] Deore B., Chen Z., Nagaoka T. (2000). Potential-induced enantioselective uptake of amino acid into molecularly imprinted overoxidized polypyrrole. Anal. Chem..

[21] Ebarvia B.S., Cabanilla S., Sevilla F. (2005). Biomimetic properties and surface studies of a piezoelectric caffeine sensor based on electrosynthesized polypyrrole. Talanta.

[22] Liang H., Ling T., Rick J.F., Chou T. (2005). Molecularly imprinted electrochemical sensor able to enantroselectivly regonize D and L-tyrosine. Anal. Chim. Acta.

[23] Ramanaviciene A., Finkelsteinas A., Ramanavicius A. (2006). Basic electrochemistry meets nanotechnology: Elelectrochemical preparation of artificial receptors based on nanostructured conducting polymer, polypyrrole. J. Chem. Educ..

[24] Özcan L., Şahin Y. (2007). Determination of paracetamol based on electropolymerized-molecularly imprinted polypyrrole modified pencil graphite electrode. Sens. Actuat. B.

[25] Özcan L., Şahin M., Şahin Y. (2008). Electrochemical preparation of a molecularly imprinted polypyrrole-modified pencil graphite electrode for determination of ascorbic acid. Sensors.

[26] Majidi M.R., Jouyban A., Asadpour-Zeynali K. (2006). Voltammetric behavior and determination of isoniazid in pharmaceutical by using overoxidized polypyrrole glassy carbon modified electrode. J. Electroanal. Chem..

[27] Ramanaviciene A., Ramanavicius A. (2004). Molecularly imprinted polypyrrole-based synthetic receptor for direct detection of bovine leukemia virus glycoproteins. Biosens. Bioelectron..

[28] Mostany J., Scharifker B.R. (1997). Impedance spectroscopy of undoped, doped and overoxidized poypyrrole films. Synth. Met..

[29] Ersöz A., Gavalas V.G., Bachas L.G. (2002). Potentiometric behavior of electrodes based on overoxidized polypyrrole films. Anal. Bioanal.Chem..

[30] Tamer U., Ertaş N., Udum Y.A., Şahin Y., Pekmez K., Yıldız A. (2005). Electrochemically controlled solid-phase microextraction (EC-SPME) based on overoxidized sulfonated polypyrrole. Talanta.

[31] Şahin Y., Ercan B., Şahin M. (2008). In situ electrochemical solid-phase extraction of anions and cations using polypyrrole and overoxidized sulfonated polypyrrole. Talanta.

[32] Şahin M., Şahin Y., Özcan A. (2008). Ion chromatography-potentiometric detection of inorganic anions and cations using polypyrrole and overoxidized polypyrrole electrode. Sens. Actuat. B.

[33] Özcan L., Şahin Y., Türk H. (2008). Non-enzymatic glucose biosensor based on overoxidized polypyrrole nanofiber electrode modified with cobalt (II) phthalocyanine tetrasulfonate. Biosens. Bioelectron..

[34] Hsueh C., Brajter-Toth A. (1994). Electrochemical preparation and analytical applications of ultrathin overoxidized polypyrrole films. Anal.Chem..

